# Supplementary material for: DDA1, a novel factor in transcription-coupled repair, modulates CRL4CSA dynamics at DNA damage-stalled RNA polymerase II
Source: Res Sq. 2023 Oct 12:rs.3.rs-3385435. Preprint. [Version 1] doi: 10.21203/rs.3.rs-3385435/v1 (PMC10602077; doi:10.21203/rs.3.rs-3385435/v1)

Supplementary Fig. 1

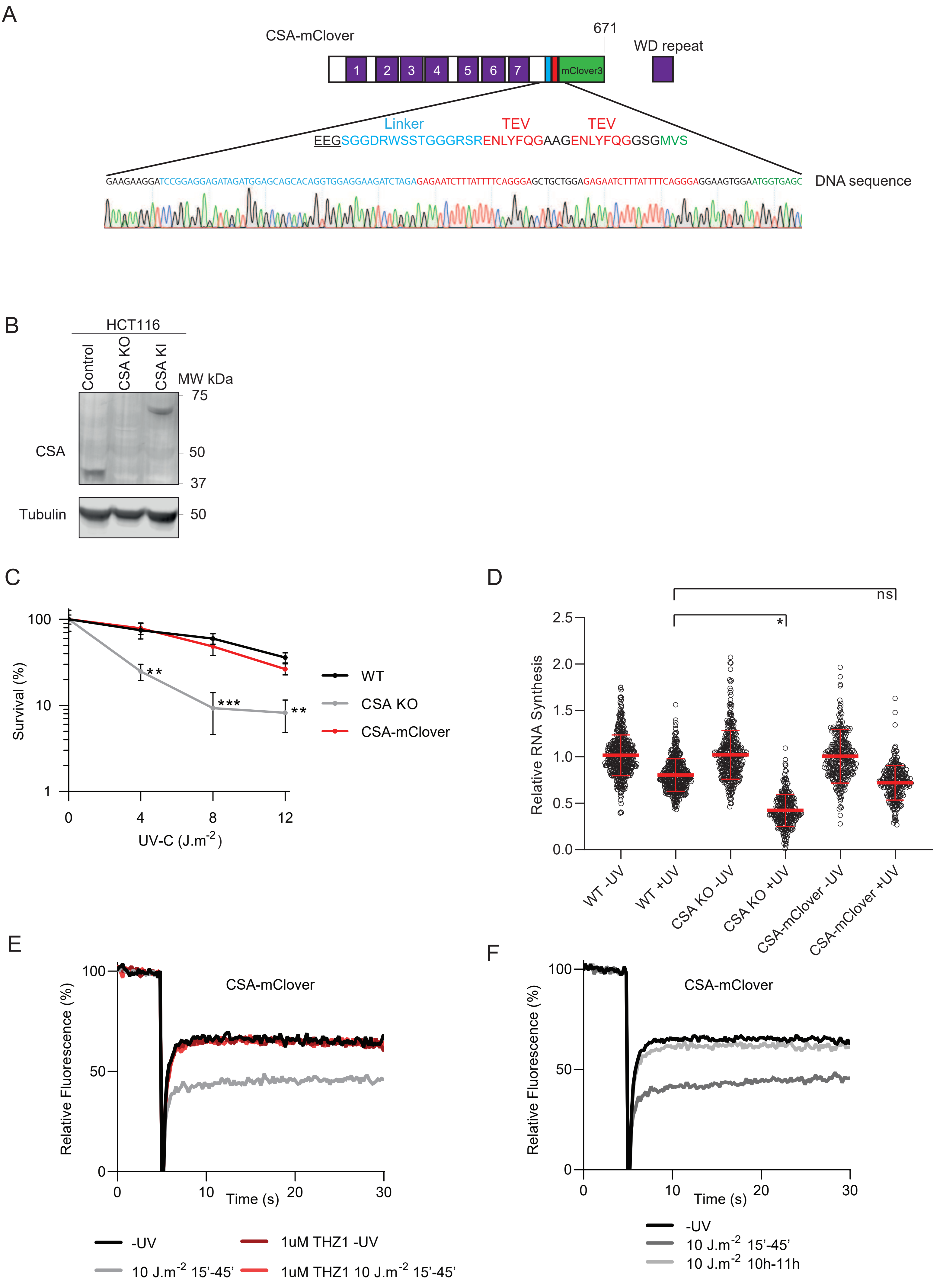

Supplementary Fig. 2

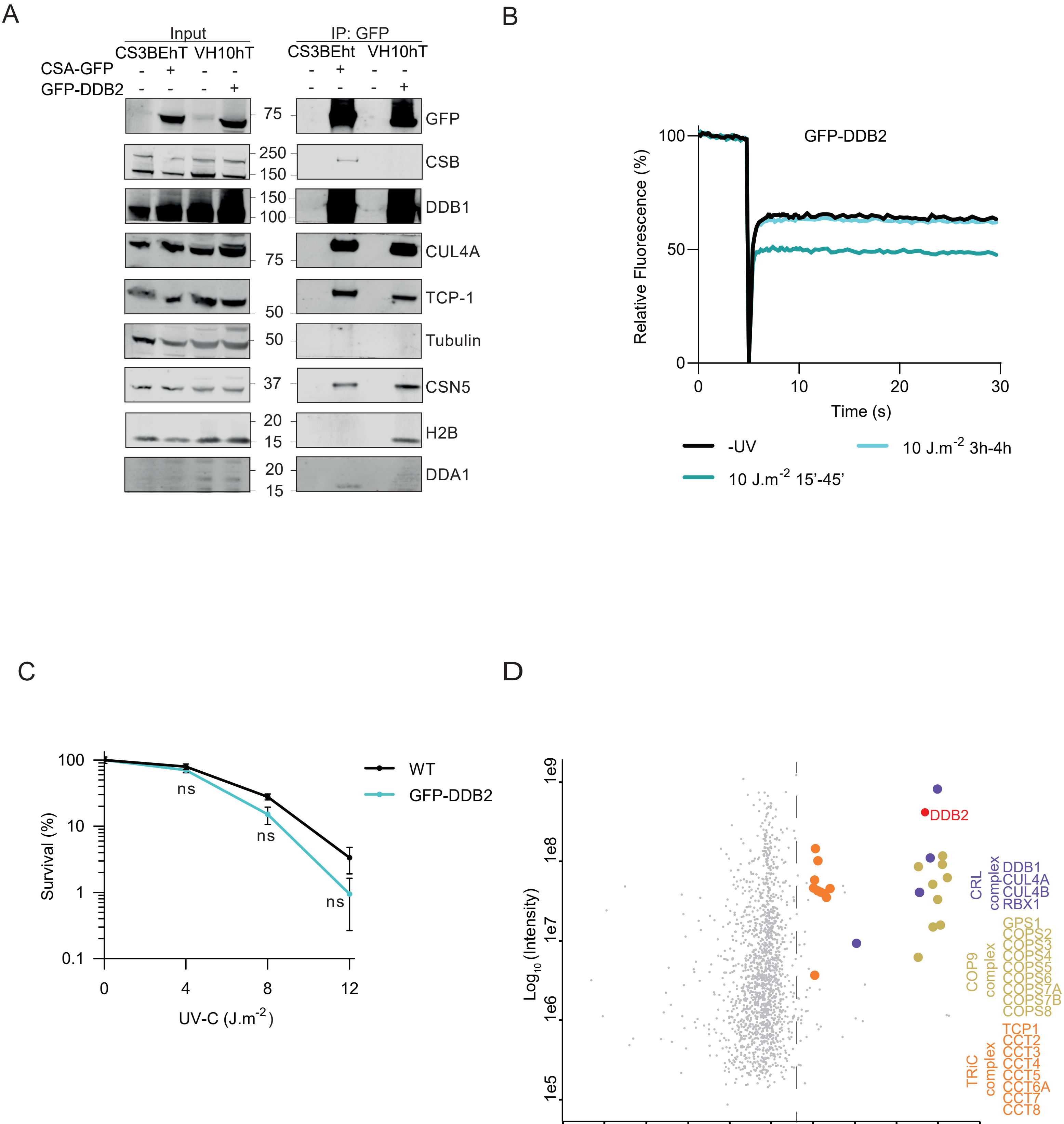

Supplementary Fig. 3

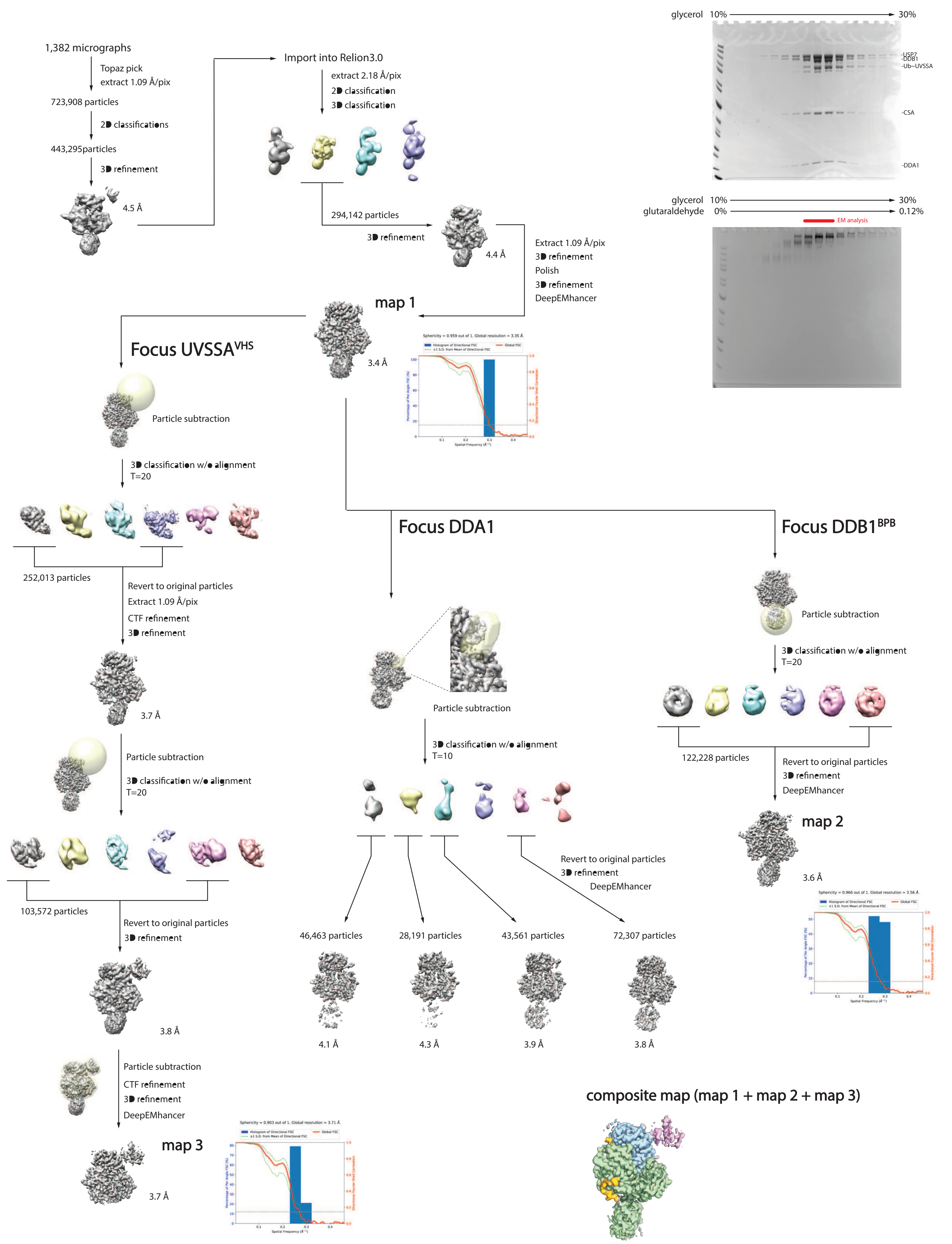

Supplementary Fig. 4

A

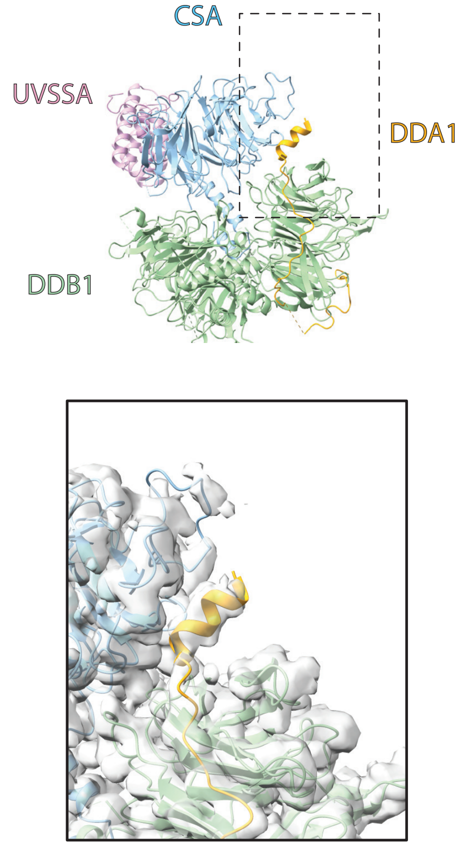

B

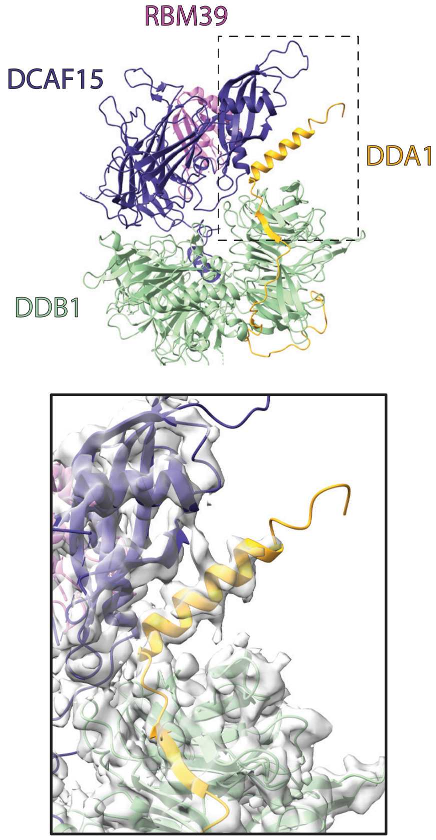

C

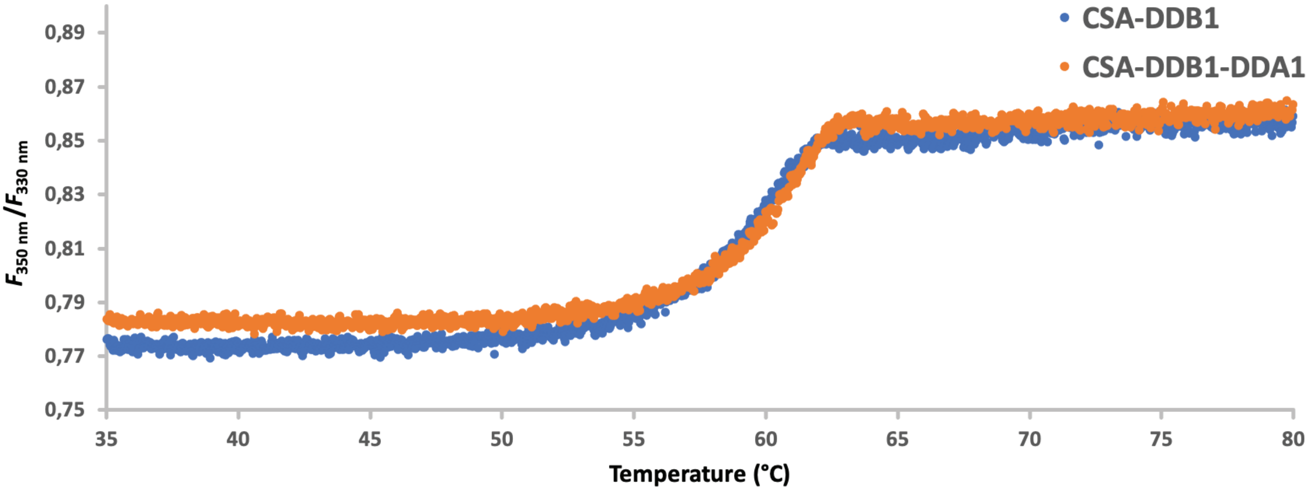

D

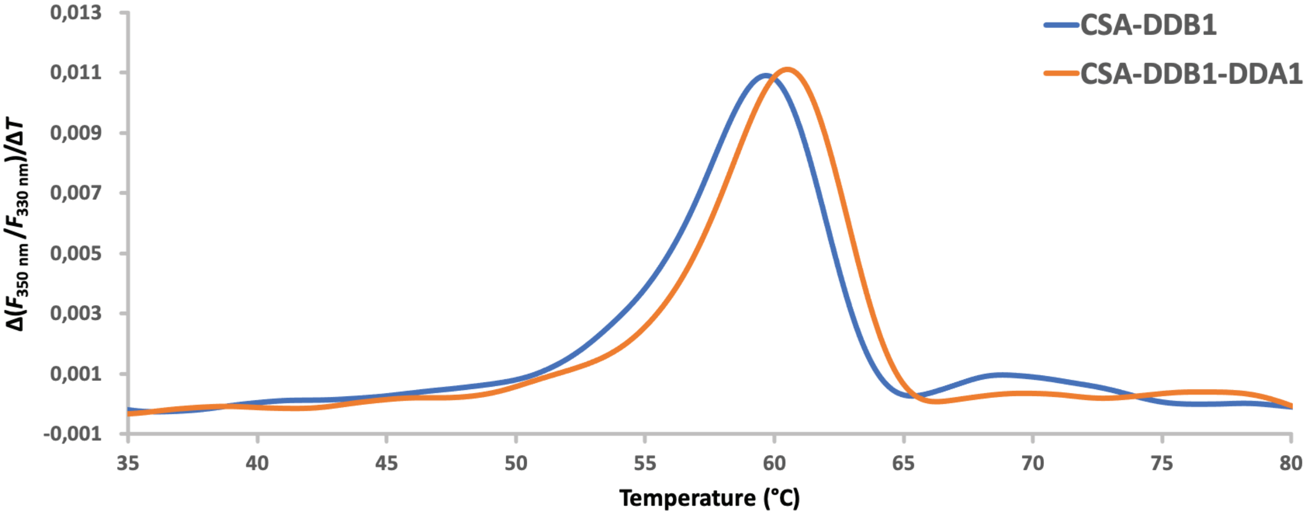

Supplementary Fig. 5

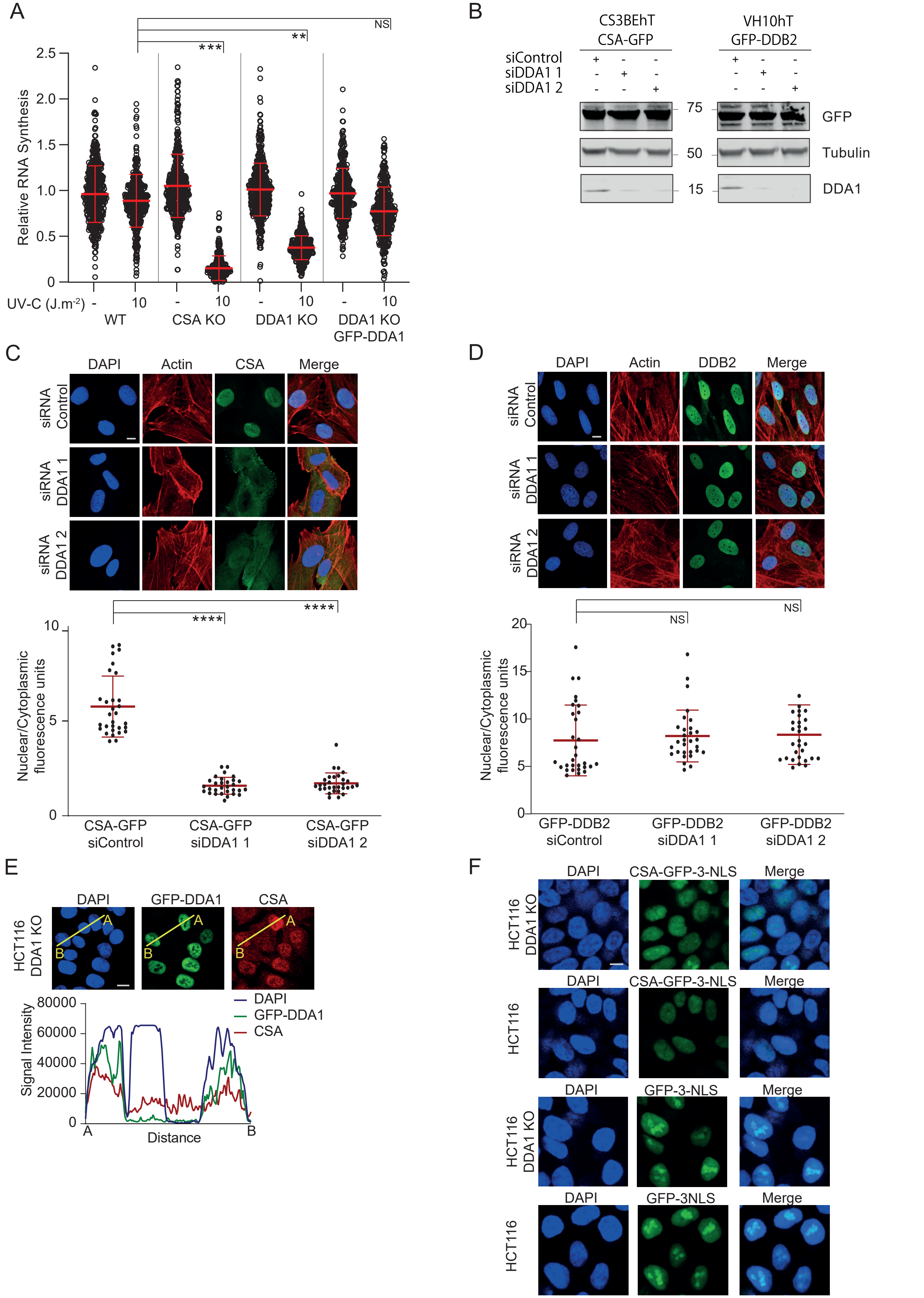

Supplementary Fig. 6

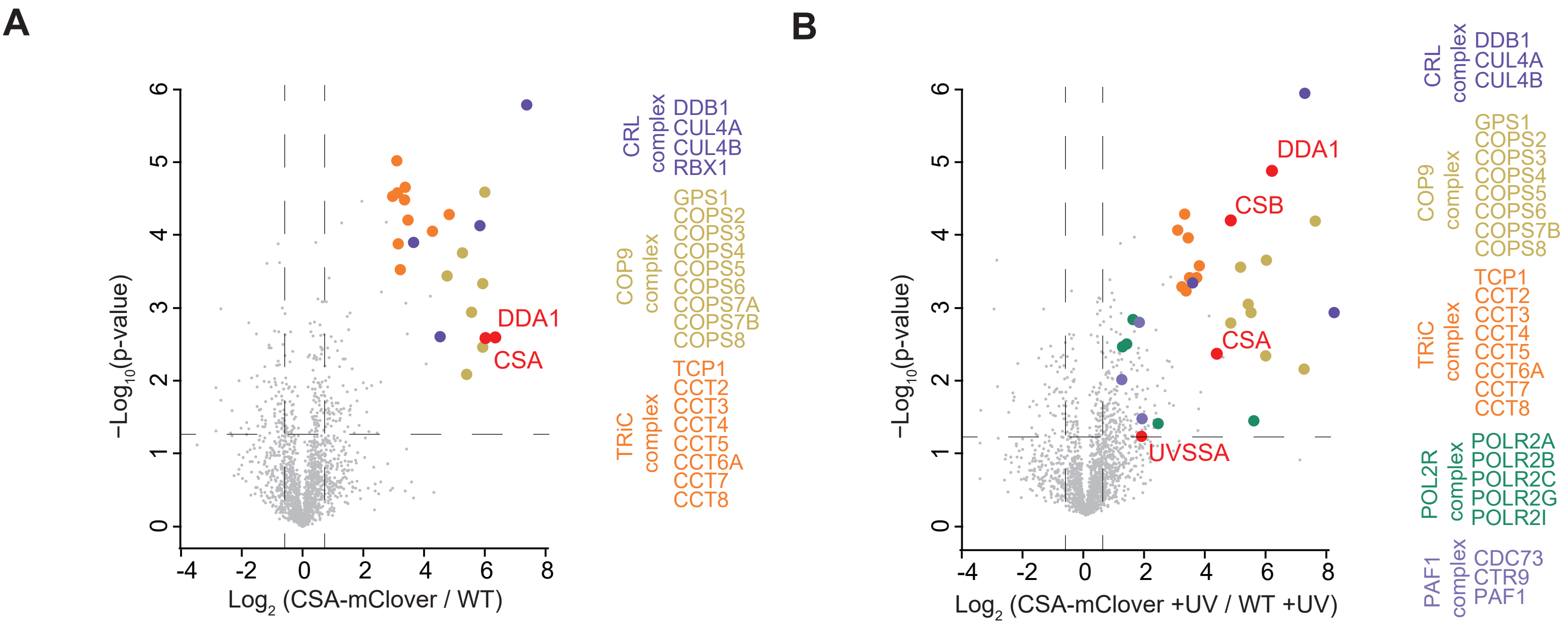

C

			CSAmClover/DDA1KO vs CSAmClover t-test Difference (Log₂)	CSAmClover/DDA1KO +UV vs CSAmClover +UV t-test Difference (Log₂)
	CSA (ERCC8)	→	**-0.175833**	**0.594915**
	DDB1	→	0.384607	0.464457
	DDA1	↓	-3.23592	-3.68452
TRiC complex	TCP1	→	0.211027	0.454005
TRiC complex	CCT2	→	0.190139	0.436647
TRiC complex	CCT3	→	0.215641	0.336879
TRiC complex	CCT4	→	0.121384	0.393906
TRiC complex	CCT5	→	0.222755	0.31822
TRiC complex	CCT6A	→	0.220267	0.274117
TRiC complex	CCT7	→	0.251577	0.372348
TRiC complex	CCT8	→	0.1789	0.344252

Supplementary Fig. 7

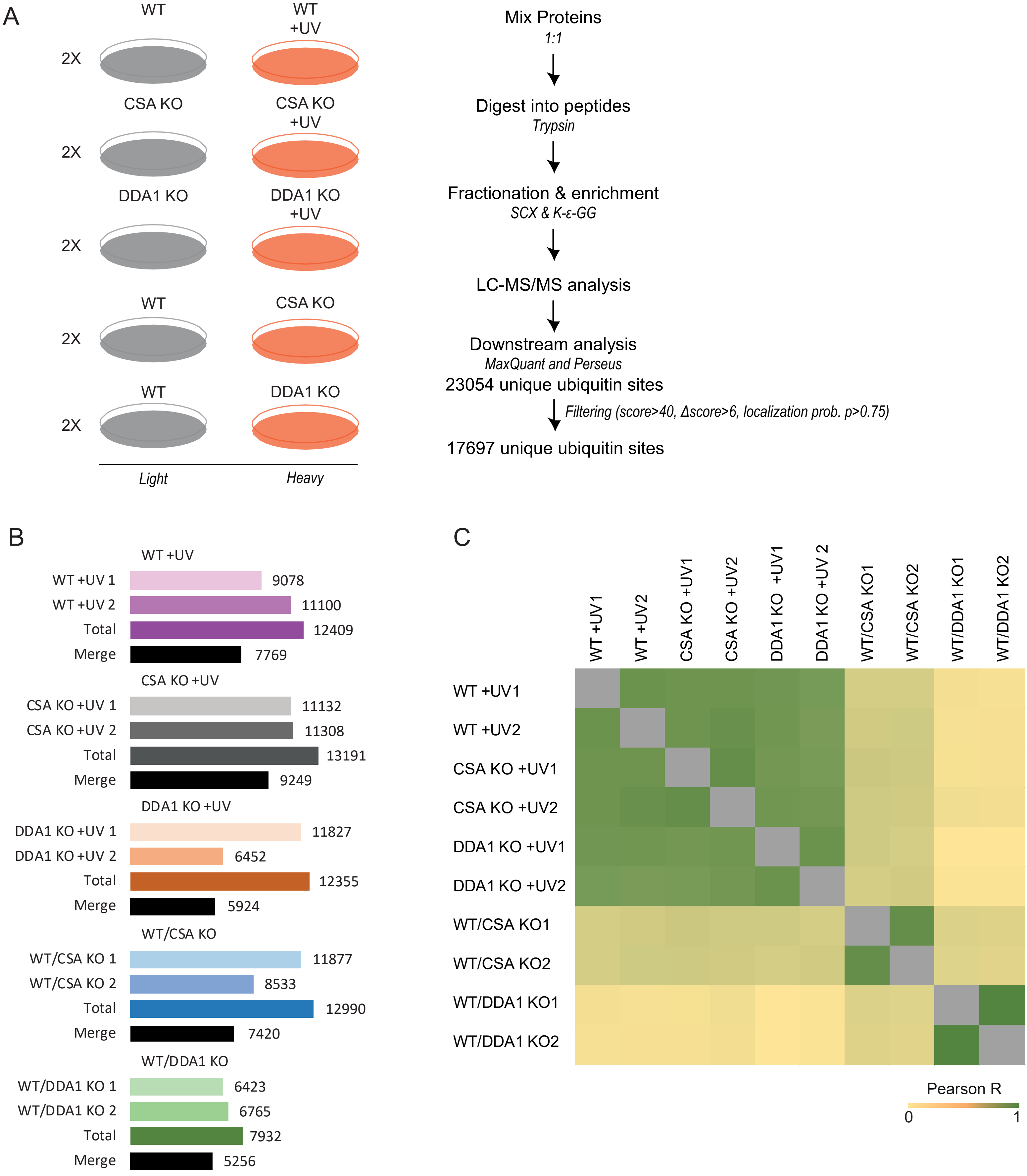

Supplementary Fig. 8

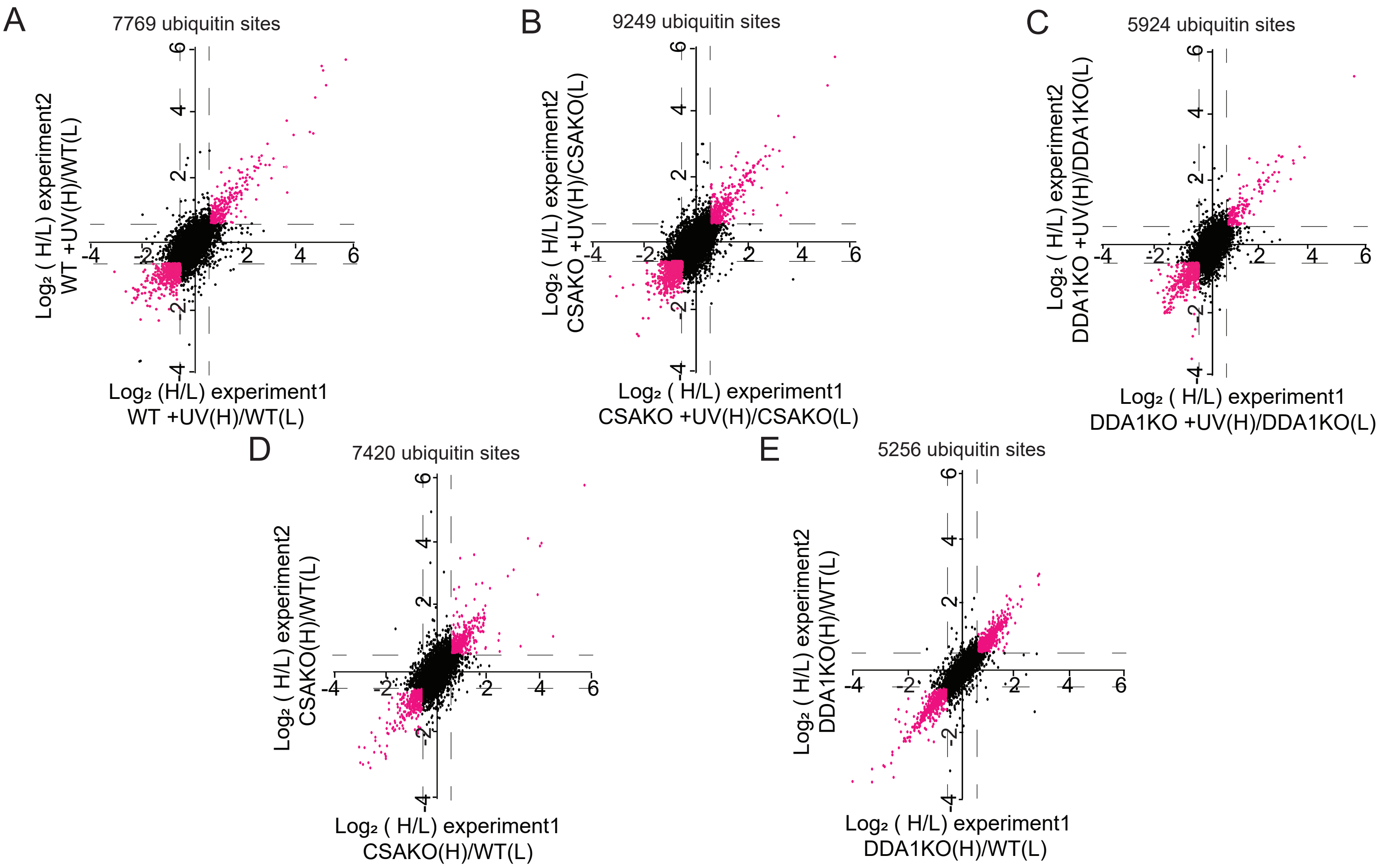

Supplementary Fig. 9

A

Top 50 up-regulated ubiquitin sites (WT)

GENE NAME (position)	Ratio H/L (Log_2) Experiment1	Experiment2
XPC (364)	5.65	5.58
ERCC2 (751)	4.91	4.80
SF3B1 (1014)	4.80	5.26
XPC (374)	4.75	5.39
XPC (359)	4.50	4.43
HELLS (204)	4.42	3.32
TAP2 (245)	4.30	3.37
TRIP4 (245)	3.70	3.29
PSMA2 (171)	3.45	1.53
PSMC1 (413)	3.43	3.72
TMA7 (30)	3.40	2.31
HSPA8 (500)	2.93	2.56
YBX1 (118)	2.86	1.96
POLR2A (1268)	2.78	2.37
ACLY (1077)	2.71	3.01
POLR2A (1350)	2.64	2.36
SMARCA5 (814)	2.50	2.65
DDB2 (309)	2.45	2.40
XPC (174)	2.40	2.60
DDB2 (362)	2.39	2.51
OLA1 (190)	2.35	2.27
MYO10 (918)	2.35	1.75
CNBP (103)	2.28	2.29
RPS16 (4)	2.22	2.18
RPS12 (129)	2.22	2.32
CUEDC2 (272)	2.14	1.95
YBX3 (96)	2.12	2.62
YBX1 (64)	2.12	2.62
SND1 (513)	2.04	1.99
RPS27A (152)	1.96	2.06
RPL12 (48)	1.95	2.22
USP48 (551)	1.95	1.77
SUPT6H (306)	1.94	1.62
POLR2A (177)	1.91	1.89
RPS3 (230)	1.89	1.97
ABCE1 (397)	1.89	1.63
RPS10 (139)	1.85	1.84
XPC (161)	1.79	1.98
CNBP (8)	1.78	1.74
TIA1 (79)	1.74	2.14
UVSSA (414)	1.74	2.55
TOP1 (642)	1.73	1.50
PHRF1 (556)	1.70	1.57
CSDE1 (682)	1.68	2.14
ASCC3 (198)	1.67	1.95
CSDE1 (288)	1.67	1.53
RNH1 (46)	1.62	2.20
PHRF1 (768)	1.56	1.69
RNF170 (3)	1.56	1.50
CLPTM1 (460)	1.55	1.79

B

Top 50 up-regulated ubiquitin sites (CSA KO)

GENE NAME (position)	Ratio H/L (Log_2) Experiment1	Experiment2
SF3B1 (1014)	5.42	5.68
XPC (374)	5.12	4.82
DSP (1033)	3.81	3.23
SH2B3 (373)	3.53	1.52
HSPA8 (500)	3.38	2.33
YBX1 (118)	3.25	2.81
PSMC1 (413)	3.21	3.88
DDB2 (309)	3.00	2.85
SMARCA5 (814)	2.86	2.65
CNBP (103)	2.77	2.25
DDB2 (106)	2.72	2.64
XPC (174)	2.71	2.96
SUPT6H (306)	2.61	2.36
SIVA1 (41)	2.57	1.68
OLA1 (190)	2.53	2.53
YBX3 (96)	2.44	2.48
YBX1 (64)	2.44	2.48
POLR2A (1350)	2.41	2.41
EDF1 (98)	2.28	2.15
RPS16 (4)	2.27	2.17
PRR14L (1861)	2.26	1.89
USP48 (551)	2.26	2.36
SUPT5H (258)	2.24	2.29
RPS12 (129)	2.24	2.36
MYH9 (856)	2.21	1.78
SPATS2L (11)	2.19	1.83
CNBP (8)	2.15	1.86
RPS10 (139)	2.14	2.16
SND1 (513)	2.12	2.21
XPC (161)	2.12	1.95
POLR2A (163)	2.09	2.15
POLR2A (1268)	2.09	1.90
CSDE1 (682)	2.03	1.88
RPL12 (48)	2.02	2.11
ACLY (1077)	2.01	2.43
ASCC3 (78)	1.99	2.44
RPS19 (111)	1.99	1.90
EIF2S2 (276)	1.96	2.04
BAZ2A (687)	1.96	2.11
POLR2A (177)	1.96	2.07
RPS3 (230)	1.91	2.00
PLEC (3106)	1.91	1.59
CUEDC2 (272)	1.86	2.06
POLH (709)	1.85	2.23
DCBLD2 (391)	1.82	1.85
TOP1 (642)	1.81	1.77
TOP1 (347)	1.80	1.61
SYNCRIP (297)	1.74	1.70
PHRF1 (768)	1.73	1.76
ASCC3 (213)	1.73	1.92

C

Top 50 up-regulated ubiquitin sites (DDA1 KO)

GENE NAME (position)	Ratio H/L (Log_2) Experiment1	Experiment2
SF3B1 (1014)	5.21	5.16
PSMC1 (413)	3.38	2.68
TAP2 (245)	3.21	3.02
SMARCA5 (814)	2.99	2.51
XPC (183)	2.97	2.59
EDRF1 (694)	2.88	2.02
POLR2A (1268)	2.57	2.71
XPC (174)	2.53	2.70
POLR2A (1350)	2.44	2.24
OLA1 (190)	2.44	2.75
HSPA8 (500)	2.38	1.95
YBX3 (93)	2.33	2.57
YBX1 (64)	2.33	2.57
RPL12 (48)	2.28	2.24
ARIH1 (293)	2.27	1.66
RNF219 (502)	2.20	2.15
CSDE1 (682)	2.07	2.07
RPS10 (139)	2.05	1.96
SYNCRIP (407)	2.04	2.46
POLR2A (177)	2.00	1.99
DDB2 (106)	1.96	1.97
XPC (161)	1.90	1.85
USP48 (551)	1.86	2.22
POLR2A (163)	1.83	1.65
FASN (1752)	1.82	1.59
CNBP (8)	1.78	1.35
PHRF1 (556)	1.78	1.75
RPS3 (230)	1.75	1.79
RPS16 (4)	1.75	1.96
GAR1 (150)	1.72	1.65
YBX3 (150)	1.72	2.00
CSDE1 (288)	1.72	1.85
SUPT6H (306)	1.70	1.97
TMA7 (30)	1.68	1.96
UVSSA (414)	1.64	2.54
RPS2 (58)	1.53	1.71
ABCE1 (397)	1.45	1.44
ASCC3 (319)	1.37	1.04
NUMA1 (1475)	1.36	2.65
DDB1 (204)	1.33	1.25
H1F0 (12)	1.28	1.19
RPS27A (113)	1.26	1.39
SMARCA5 (836)	1.26	1.26
SUPT6H (1100)	1.26	1.83
DRG1 (46)	1.25	1.19
PSIP1 (6)	1.24	1.39
RPS3 (214)	1.23	1.34
TANK (199)	1.23	1.19
RPA1 (167)	1.21	1.11
SND1 (886)	1.17	1.36

Supplementary Fig. 10

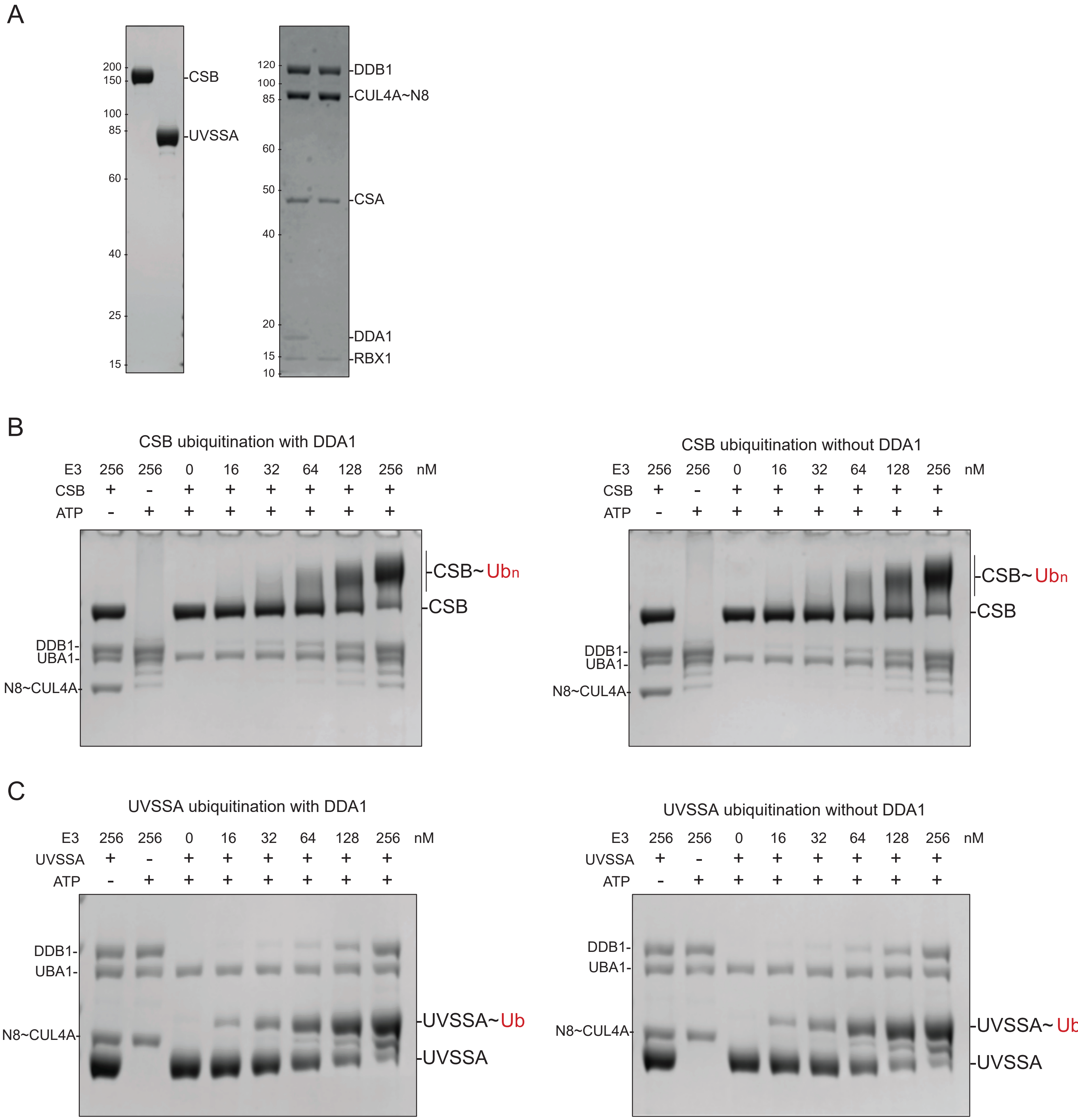

Supplementary Fig. 11

A

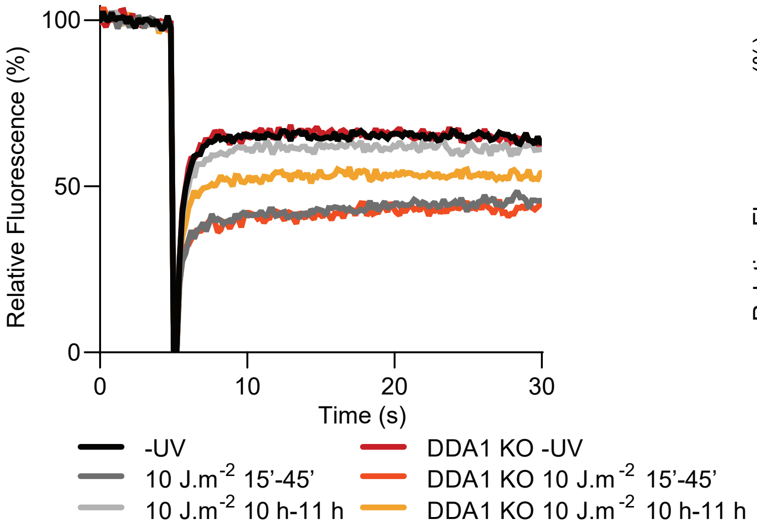

B

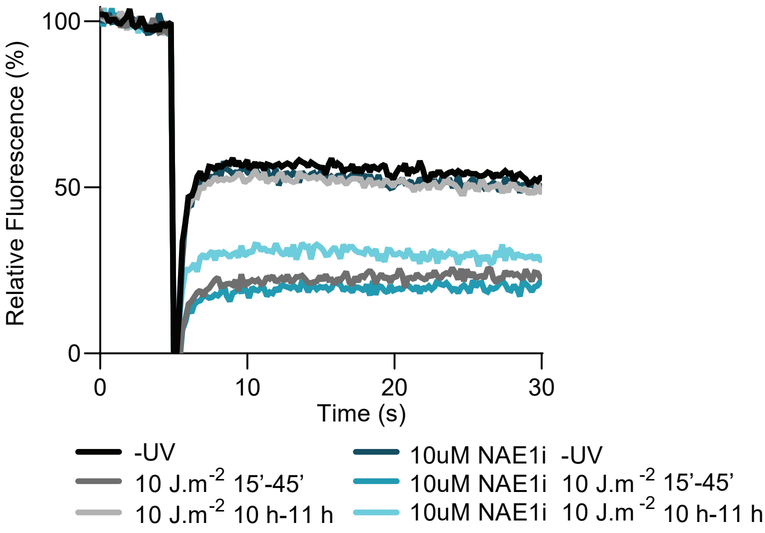

Supplementary Fig. 12

Figure 1C

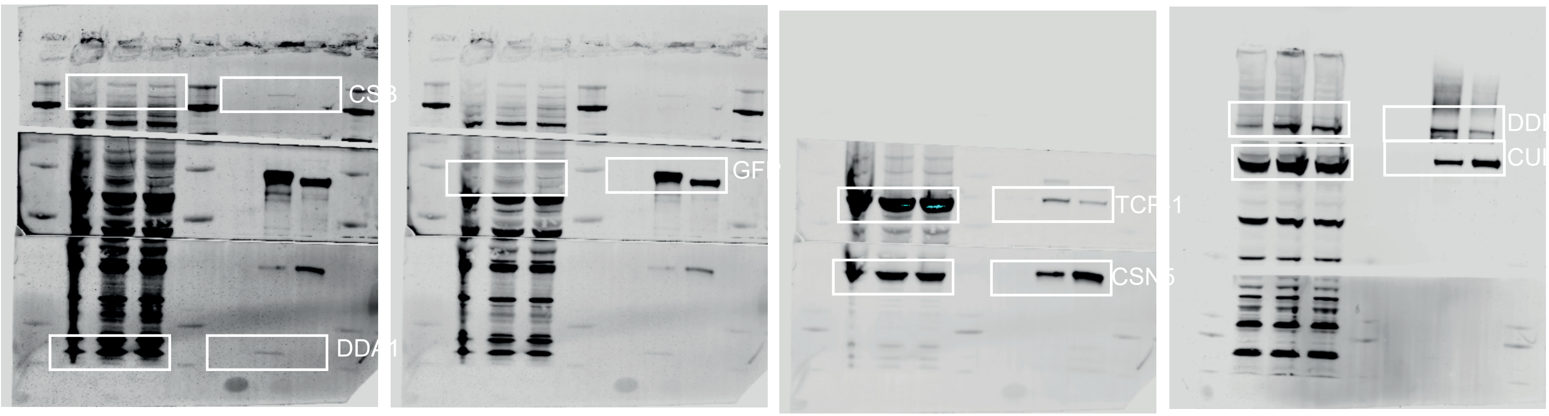

Figure 3C

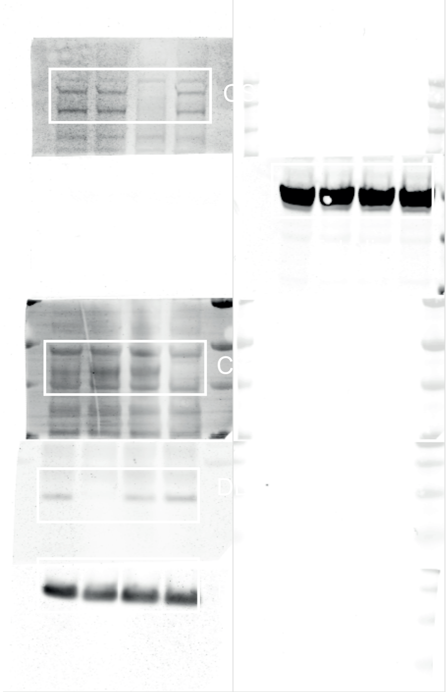

Supplementary Figure 1B

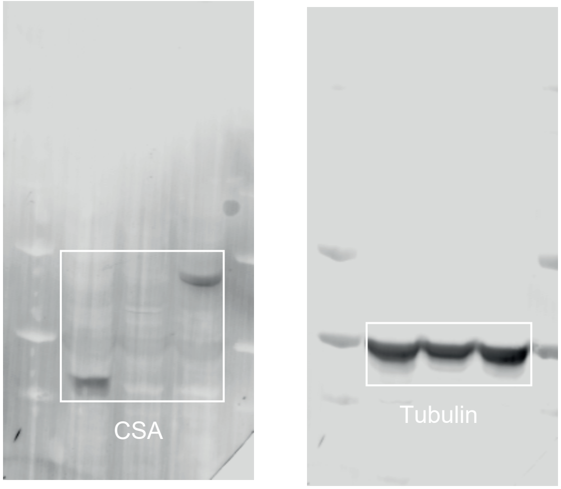

Supplementary Figure 2A

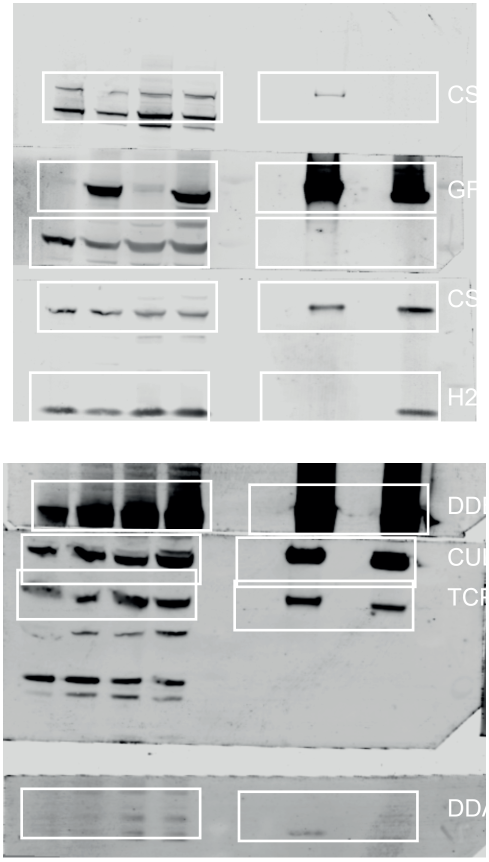

Supplementary Figure 3C

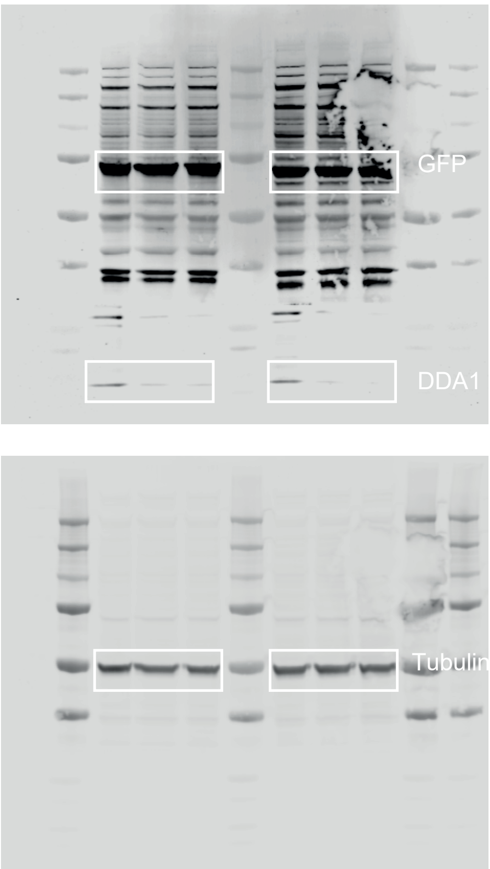

Figure 6C

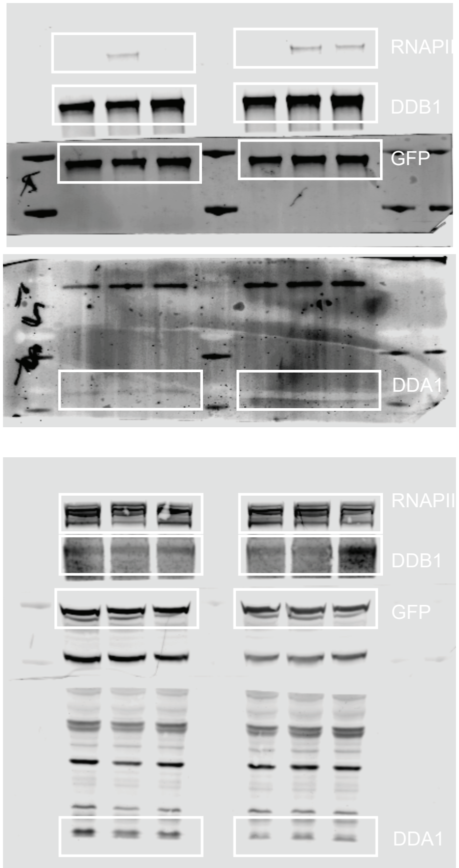

Supplementary Fig 10B

with DDA1

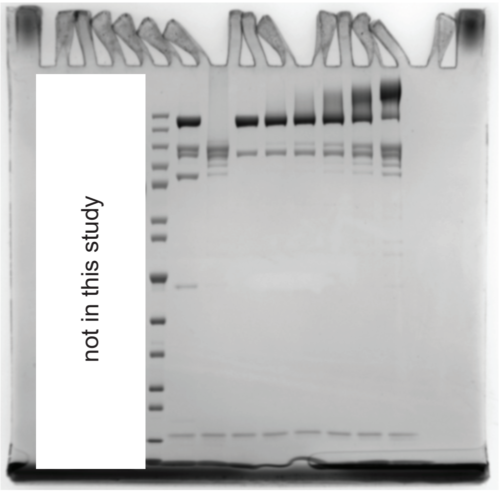

without DDA1

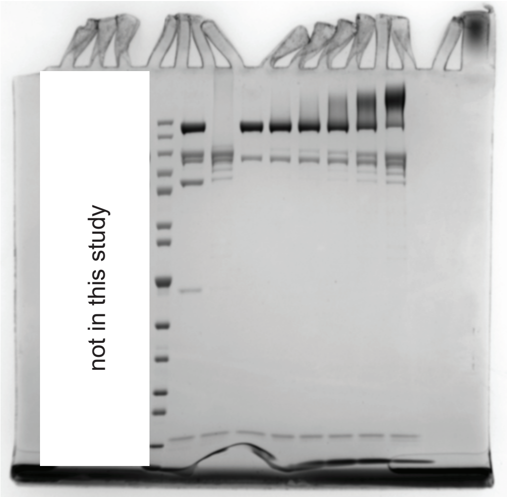

Supplementary Fig 10C

with DDA1

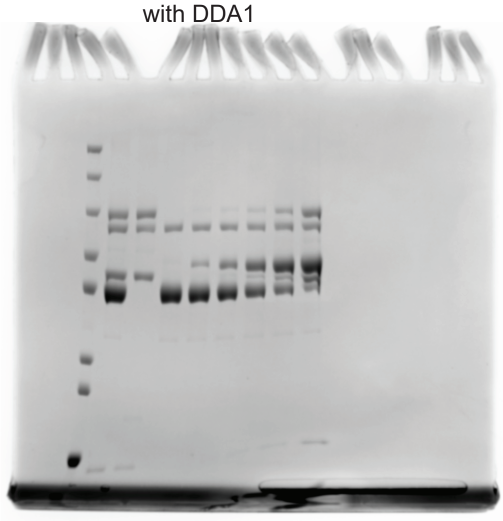

without DDA1

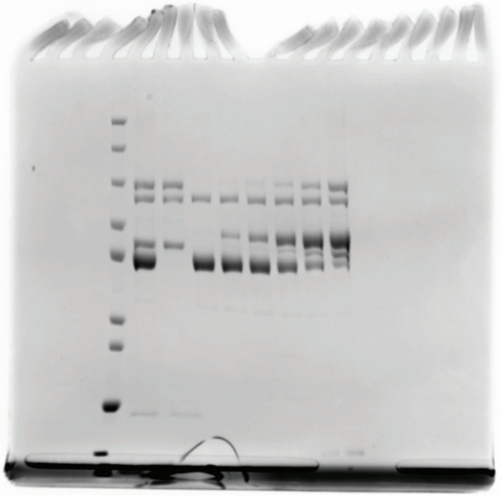

Supplement: Supplement 1 — Supplementary Figure 1. A, Schematic representation of the CSA-mClover, which includes a linker and two TEV protease recognition sequences. B, Immunoblot of indicated HCT116 cell lines showing CSA or CSA-mClover expression. Tubulin was used as loading control. C, Relative colony survival of the indicated HCT116 WT, CSA-mclover KI and CSA KO cells exposed to the indicated doses of UV. Data shown represent the mean ± SD from three independent experiments. D, Transcription restart after UV damage as determined by relative EU incorporation in the indicated HCT116 WT, CSA-mClover KI and CSA KO cells, at 24 h after UV exposure (10 J.m−2) or mock treated. RNA synthesis was measured by EU incorporation and levels were normalized to the non-irradiated cells (set to 1) and each normalized EU signal is shown as one data point. The mean ± S.D. is indicated in red from three independent experiments. E, Fluorescence Recovery After Photobleaching (FRAP) analysis of CSA-mClover mobility in presence or absence of THZ1inhibitor added 1 h before irradiation and followed by UV irradiation (10 J.m−2). Graphs depict the mean & S.E.M. of ≥ 30 cells from at least three independent experiments. F, FRAP analysis of CSA-mClover in mock and UV treated (10 J.m−2) (the data are reported also in Supplementary Figure 13A). Graphs depict the mean & S.E.M. of ≥ 30 cells from at least three independent experiments. Data shown in C represent NS, *P ≤ 0.05, **P ≤ 0.01, ***P ≤ 0.001 relative to WT analysed by unpaired, two-tailed t-test, adjusted for multiple comparison. Data shown in D represent NS, *P ≤ 0.05 analysed by a nested t-test. Supplementary Figure 2. A, IP of CSA and DDB2 using GFP beads from CSA-GFP expressed in CS3BEhT and GFP-DDB2 expressed in VH10hT cells followed by immunoblotting for the indicated proteins. CS3BEhT and VH10hT cells were used as a control. B, FRAP analysis of GFP-DDB2 mobility from GFP-DDB2 KI HCT116 1 and 3 h after UV irradiation (10 J.m−2). Graphs depict the mean & S. [file NIHPPrs3385435v1-supplement-1.pdf]
