# Supplementary material for: DDA1, a novel factor in transcription-coupled repair, modulates CRL4CSA dynamics at DNA damage-stalled RNA polymerase II
Source: Res Sq. 2023 Oct 12:rs.3.rs-3385435. Preprint. [Version 1] doi: 10.21203/rs.3.rs-3385435/v1 (PMC10602077; doi:10.21203/rs.3.rs-3385435/v1)
Supplement: Supplement 2 [file NIHPPrs3385435v1-supplement-2.pdf]

Supplementary Table 1
Table with SILAC ratios as determined using quantitative interaction proteomics (CSAmClover).

Supplementary Table 2
Table with SILAC ratios as determined using quantitative interaction proteomics (GFP-DDB2).

Supplementary Table 3
Table label free (Data Independent Analysis) as determined using quantitative interaction proteomics (CSAmClover in WT and DDA1KO cell lines).

Supplementary Table 4
Table with SILAC ratios as determined using quantitative proteomics of ubiquitin peptides (WT, CSAKO, DDA1KO HCT116 cell lines).

Supplementary Table 5
Table with SILAC ratios as determined using quantitative proteomics of total proteins (WT, CSAKO, DDA1KO HCT116 cell lines).
